# Hemophagocytic lymphohistiocytosis secondary to disseminated histoplasmosis in an HIV-negative patient: A case of misdiagnosis

**DOI:** 10.1016/j.mmcr.2025.100752

**Published:** 2025-11-19

**Authors:** Hongzhen Shu, Xiaofeng Li, Yuying Chen, Weihong Wang

**Affiliations:** a Department of Infectious Diseases, Huzhou Central Hospital, Affiliated Central Hospital Huzhou University, Huzhou, Zhejiang Province, 313000, China; b Huzhou Key Laboratory of Precision Medicine Research and Translation for Infectious Diseases, Zhejiang Province, 313000, China; c Department of Infectious Diseases, First Affiliated Hospital of Huzhou University, Huzhou, Zhejiang Province, 313000, China

**Keywords:** Hemophagocytic lymphohistiocytosis, Disseminated histoplasmosis, *Histoplasma capsulatum*, HIV seronegativity, Misdiagnosis

## Abstract

Hemophagocytic lymphohistiocytosis (HLH) secondary to disseminated histoplasmosis (DH) is rare and often misdiagnosed, especially in non-endemic areas. We present a case of a 70-year-old Chinese man who was admitted with fever, nausea, and vomiting, initially misdiagnosed with pulmonary tuberculosis. The use of metagenomic next-generation sequencing (mNGS) played a crucial role in the early and accurate diagnosis, highlighting its potential as a valuable diagnostic tool for rare infections.

## 1 Introduction

Histoplasmosis is an invasive fungal disease (IFD) caused by *Histoplasma capsulatum* that is endemic mainly in the United States [1], and sporadically in Asia [2]. It mainly involves the lung, with uncommon disseminated form [3]. Histopathological examination and/or fungal culture is the gold standard for the diagnosis [4]. The detection of Histoplasma capsulatum antigen in serum and urine demonstrates high positivity, especially in disseminated cases where urine antigen exceeds 90 %, making it a valuable tool for early diagnosis and therapeutic monitoring [5]. Disseminated histoplasmosis (DH) is easily overlooked and misdiagnosed, especially in non-endemic areas. It has been reported to be misdiagnosed as pulmonary tuberculosis (TB), leishmaniasis or lymphoma and Crohn's disease and sarcoidosis [6]. Although histoplasmosis is typically self-limited in immunocompetent individuals, severe complications such as secondary HLH may occasionally occur. Misdiagnosis or delayed diagnosis can cause high mortality. So far, five HIV-negative cases of HLH secondary to DH have been reported in China [[7], [8], [9], [10], [11]]. In recent years, metagenomic next-generation sequencing (mNGS) is of great value in the early diagnosis of rare infectious diseases due to its unique sensitivity to pathogens [12]. Here we reported a misdiagnosed case of HLH due to DH in an HIV-negative Chinese patient and the corrected diagnosis was rapidly made by the mNGS.

## 2 Case presentation

On October 26, 2022, a 70-year-old Chinese man was admitted to another hospital with a two-month history of recurrent cough and sputum production. During hospitalization, cytopenias were noted, and the mNGS of bronchoalveolar-lavage fluid (BALF) revealed 7787 and 1022 sequences of *Pneumocystis jirovecii* and *H. capsulatum*, respectively. The interferon-γ (IFN-γ) release assay was negative. Abdominal ultrasound revealed splenomegaly. He was discharged with a presumptive diagnosis of pulmonary tuberculosis and subsequently started on anti-tuberculosis therapy.

On November 17, 2022, this patient presented to our hospital with fever, anorexia, nausea and vomiting for half a month. He denied weight loss, dizziness, or swollen lymph nodes. The patient reported long-term exposure to a damp and poorly lit living environment, and denied any history of autoimmune diseases or acquired immune deficiency syndrome. Upon physical examination, the liver and spleen were not enlarged.

After admission to our hospital, laboratory examination showed hematocytopenia, white blood cell count (WBC) 2.7 × 10^9^/L [(3.5–9.5) × 10^9^/L], red blood cell count (RBC) 3.45 × 10^12^/L [ (4.30–5.80) × 10^12^/L], hemoglobin (HB) 103.0g/L [(130.0–175.0) g/L], platelet count (PLT) 27.0 × 10^9^/L [(125.0–350.0) × 10^9^/L], and neutrophil count (N) 2.2 × 10^9^/L [(1.8–6.3) × 10^9^/L]. C-reactive protein (CRP) level was moderately high as 43.5mg/L (<10 mg/L), procalcitonin (PCT) level was 5.84ng/mL (<0.50 ng/mL), IgE quantification 264.5 IU/mL (<100.0 IU/mL), and decreased albumin (ALB) 25.6 g/L (40.0–55.0 g/L). The levels of fibrinogen, erythrocyte sedimentation rate (ESR), triglyceride (TG), alanine aminotransferase (ALT), aspartate aminotransferase (AST), and creatinine (Cr) were normal. No acid-fast bacilli were found on a sputum smear. Serologic tests for hepatitis B viruses, hepatitis C viruses, and human immunodeficiency virus (HIV) were negative. Anti-IFN-γ antibody was negative. *Treponema pallidum* particle agglutination test was positive, it may remain positive for life even after cure The titer of non-specific antibodies (RPR/TRUST) has dropped to negative for more than 6 consecutive months, while specific antibodies are positive. Combined with the medical history and re-examination results, it is considered as residual positive antibodies after syphilis treatment. Currently, there is no evidence of active infection, and re-administration of anti-syphilis treatment is not required.

**Fig. 1:**
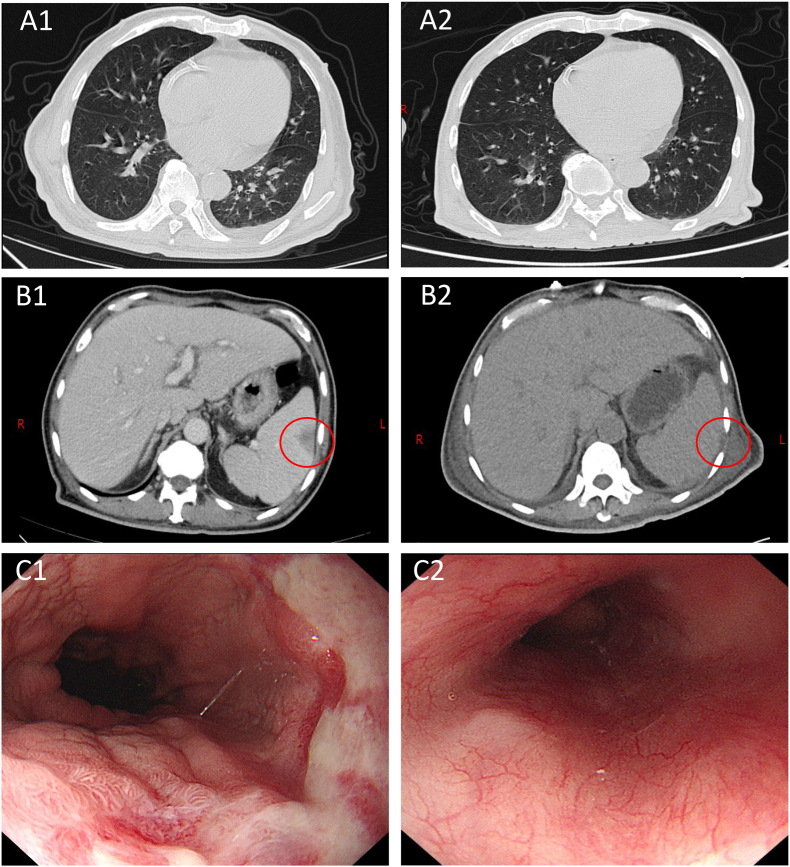
(A1-2) Changes of lung lesions before and after treatment; (B1-2) Changes of small splenic infarction before and after treatment (red circle); (C1-2) Changes of esophageal fungal infection before and after treatment.

**Fig. 2:**
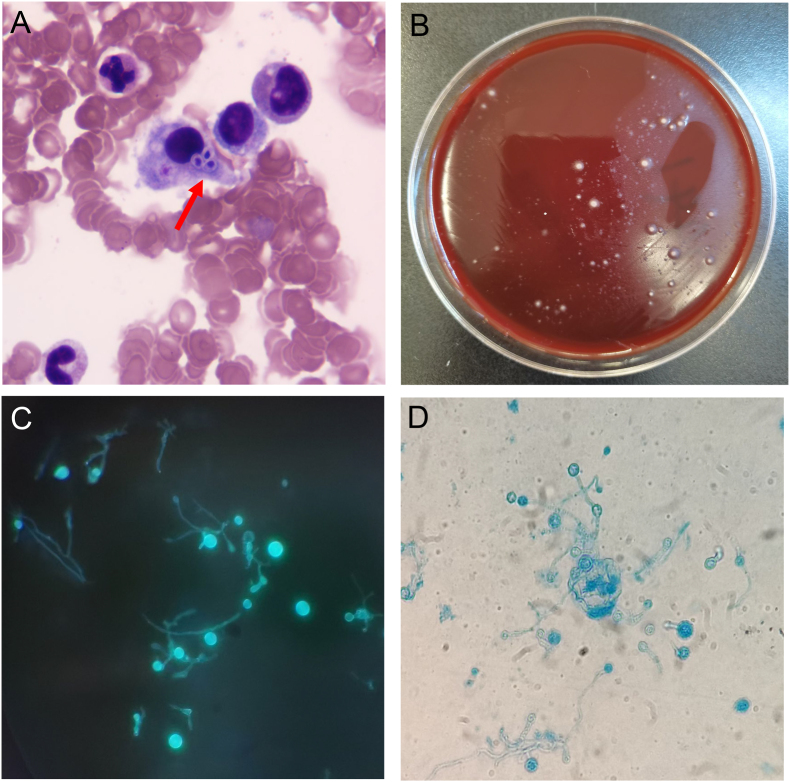
(A) Wright's stain of bone marrow smear (1000 × ) and yeast-like fungi are indicated by an arrow; (B) The colonies of *H. capsulatum* on blood culture dish after 4 weeks at 25 °C; (C) Hyphae and spores are bright blue fluorescent in Fluorescence staining (400 × ); (D) Cogwheel-like macroconidia of Histoplasma capsulatum stained with Lactophenol cotton blue (400 × ).

**Fig. 3:**
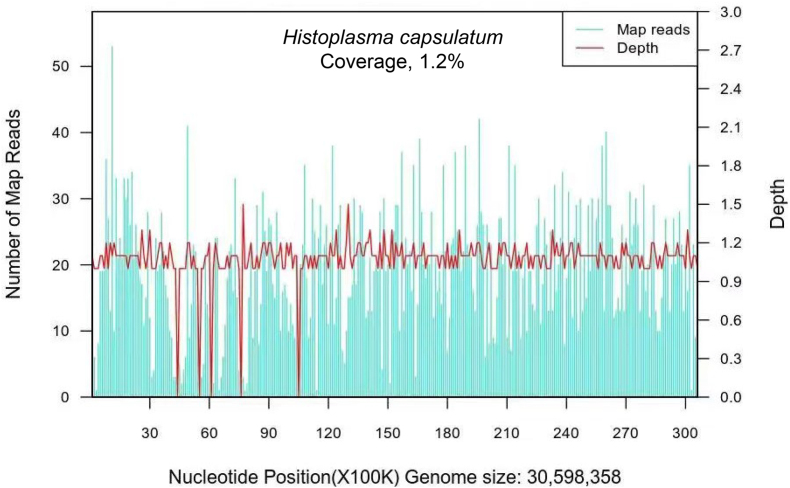
The reads number at various nucleotide position in Histoplasma capsulatum species (genome size 30,598,358) is shown by blue lines, and the depth is shown by red lines.

During hospitalization, the blood cells were progressively decreased: the lowest level of the blood cells were: HB 74g/L, RBC 2.57 × 10^12^/L, PLT 5 × 10^9^/L, WBC 1.6 × 10^9^/L, N 1.3 × 10^9^/L [ (1.8–6.3) × 10^9^/L]. The lowest level of ALB was 20.3g/L and fibrinogen was 1.37g/L. The percentage of NK cells was within the normal range. TG (triglyceride), ALT, AST, and Cr remained within normal limits, and autoimmune tests were negative. On the second day, Chest CT showed scattered small nodular lesions in both lungs (Fig. 1 A1). Abdominal CT revealed no splenomegaly, and the presence of small infarcts in the spleen (Fig. 1 B1). Due to the unexplained high fever, hematocytopenia and splenomegaly, a bone marrow aspiration and smear were performed to help us to rule out hematologic diseases. On the sixth day, the bone marrow smear showed hemophagocytosis (Fig. 2 A), which prompted us to consider the diagnosis of HLH. According to the HLH-2004 criteria, he fulfilled 5 of 8 diagnostic criteria: fever ≥38.5 °C, splenomegaly, ≥2 cytopenias, fibrinogen 1.37g/L (<1.5g/L), and evidence of hemophagocytosis in the bone marrow. On the eighth day, mNGS suggested *H. capsulatum* (Fig. 3). Strain identification in peripheral blood suggested infection with *H. capsulatum* var. *capsulatum*, and this pathogen was confirmed after 4 weeks of bone marrow culture at 25 °C. As shown in Fig. 2B–D, the diagnosis was HLH secondary to DH.

Upon transfer to our institution, anti-tuberculosis therapy was promptly discontinued in light of the evolving clinical picture, and supportive treatments were given. After 24 hours, the patient was still in a state of high fever, and the level of inflammatory indicators were significantly increased. Infectious fever could not be ruled out, and empirical anti-infection treatment was given after blood culture was done. For HLH, the patient was treated with intravenous immunoglobulin (400mg/kg∗d) for 5 consecutive days, and methylprednisolone (1mg/kg∗d) for 8 consecutive weeks, and converted to oral prednisone for gradual tapering after the patient's condition stabilized. Amphotericin B deoxycholate (AmB-D, initial dose 5mg, increasing daily to 25mg once daily) was injected for antifungal therapy. After 14 days of treatment, the patient received intravenous infusion of voriconazole (VCZ, 150mg twice daily) as sequential treatments and oral voriconazole was continued after discharge. On December 27, 2022, esophageal fungal infection was found by outpatient gastroscopy. *H. capsulatum* was still considered, and medication was continued. The patient remained on medication until January 13, 2023. On January 18, 2023, gastroscopy showed resolution of the infection (Fig. 1 A2, B2, C1-C2). Recent clinical follow-up confirmed the patient's survival; however, his general health status requires ongoing medical monitoring.

## 3 Discussion

HLH is a rare and fatal state of immune hyperactivation dividing into two categories: primary HLH and secondary HLH. Primary HLH is usually caused by genetic defects. Secondary HLH can be induced by infection, tumor, autoimmune diseases and other diseases. Recent studies suggest that the pathogenesis of HLH is mainly due to the defective perforin dependent cytotoxicity mediated by NK cells and cytotoxic T lymphocytes [13]. The sequential “cytokine storm” results in tissue damage and organ failure [14]. The detection of cytotoxic function and HLH related gene protein expression are also the reliable examination methods [15]. In our case, *H. capsulatum* infection was mainly considered as a trigger for secondary HLH.

Histopathological examination and/or fungal culture is the gold standard for the diagnosis of histoplasmosis. But for patients with severe complications, 4–8 weeks waiting for the culture results may lead to delayed treatment. Compared with culture, histopathology is faster but less sensitive and specific [16]. In recent years, with the development of high-throughput genetic testing technology, mNGS has been widely used in the diagnosis of infectious diseases, especially for rare pathogens. mNGS is reported to be valuable for diagnosing cerebral and epiglottic histoplasmosis. [17,18]. In present study, mNGS was applied in the early stage of the disease, and its reliability was confirmed by pathogen culture. For patients with HLH secondary to DH, early and rapid diagnosis is very important for treatment and prognosis.

Most of the patients with histoplasmosis were in immunocompromised state, such as HIV infection and SLE, which is in line with the reported literature [19,20]. We summarized the reported cases in China (including this case) (Table 1). Among the six cases, none were infected with HIV and all had a good prognosis. This shows that immune function affects the prognosis of severe complications of disseminated histoplasmosis. In terms of diagnostic methods, all patients were diagnosed by bone marrow histopathology, and 4/6 had bone marrow culture. Most importantly, our case is the first case of misdiagnosis. The final diagnosis of this case was hemophagocytic lymphohistiocytosis secondary to disseminated histoplasmosis. For antifungal therapy, 3/6 were treated with AmB alone and 2/6 with AmB/Itraconazole (Itra). Our case accepted AmB-D/VCZ for antifungal infection, and the general condition improved after treatment. Since extensive clinical data on VCZ is not yet available to demonstrate its therapeutic effect statistically, we hope this case can provide a valuable foundation for future research.

**Table 1:** Clinical features of Hemophagocytic Lymphohistiocytosis Secondary to Disseminated Histoplasmosis in China: 6 cases report.

Reference	[7]	[8]	[9]	[10]	[11]	Present case
Published year	2017	2020	2021	2022	2022	–
Age	37	16	27	44	46	70
Gender	Male	Male	Male	Male	Male	Male
Occupation	A blood bank	/	/	/	A cooker	/
History of tourism	/	/	/	Kenya	/	/
Leucocytes ( × 10^9^/L)	1.86	2.35	3.45	2.2	1.6	1.6
Neutrophils ( × 10^9^/L)	/	0.84	/	1.63	1.3	1.3
Hemoglobin (g/L)	86	118	87	63	63	74
Platelet ( × 10^9^/L)	2	62	32	4	44	5
ALT (U/L)	82	32	98.6	40	53	NR
AST (U/L)	70	45	32.3	49	81	NR
LDH (U/L)	352	/	468	/	/	/
ALP (U/L)	222	93	/	/	/	/
Fibrinogen (g/L)	/	3.14	3.6	/	1	NR
Serum ferritin (ng/mL)	543.3	403.8	1900	2545	2775	/
Soluble CD25 (U/ml)	/	>44000	>7500	35854	/	/
Triglycerides (mg/mL)	/	1.44	/	/	/	NR
Hepatomegaly	+	/	+	+	+	–
Splenomegaly	+	+	+	/	+	+/−
Pulmonary nodules	/	/	+	+	/	+
Lymphadenopathy	/	/	+	/	+	–
Underlying disease	No	No	Hepatitis	No	No	No
Number of HLH-2004 criteria	5	5	6	6	6	5
Diagnosis of histoplasmosis	BM Path; Blood cultures;	BM Path; BM cultures;	BM Path;	BM Path; BM cultures; mNGS;	BM Path; BM cultures;	BM Path; BM cultures; mNGS;
Antifungal treatment	AmB	AmB	AmB	AmB/Itra	AmB/Itra	AmB/VCZ
HLH treatment	IVIG	Etoposide, MP	Etoposide, MP, LD	IVIG	IVIG/Prednisone	IVIG/MP
Outcomes	Survived	Survived	Survived	Survived	Survived	Survived

ALT: alanine aminotransferase; AST: aspartate aminotransferase; LDH: lactate dehydrogenase; ALP: alkaline phosphatase; PS: present study; NR: normal range; BM: bone marrow; Path: histopathology; mNGS: metagenomic next-generation sequencing; AmB: Amphotericin B; Itra: itraconazole; VCZ: voriconazole; IVIG: intravenous immunoglobulin; MP: methylprednisolone; LD: liposome doxorubicin.

## CRediT authorship contribution statement

**Hongzhen Shu:** Writing – original draft. **Xiaofeng Li:** Writing – review & editing. **Yuying Chen:** Visualization, Data curation. **Weihong Wang:** Writing – review & editing, Supervision, Conceptualization.

## Consent to participate

Written informed consent was obtained from the patient for publication.

Approved by the Ethics Committee of Huzhou Central Hospital (No: 202309001–01).

## Fundings

This work was supported by the Zhejiang Public Welfare Application Research Project (No. LGF22H190006), the Key Program of the Public Welfare Technology Application Research of Huzhou (Grant No. 2024GZ82), and Huzhou Key Laboratory of Precision Medicine Research and Translation for Infectious Diseases.

## Conflict of interest

There are none.
